# Alexithymia May Be Associated With Depression in Early-Stage Breast Cancer Patients

**DOI:** 10.7759/cureus.29519

**Published:** 2022-09-23

**Authors:** Pinar Eraslan, Aysegul İlhan, Cengiz Karacin, Ömür Berna Çakmak Öksüzoğlu

**Affiliations:** 1 Psychiatry, Presidential Health Center, Ankara, TUR; 2 Medical Oncology, University of Health Sciences, Dr. Abdurrahman Yurtaslan Ankara Oncology Training and Research Hospital, Ankara, TUR

**Keywords:** mastectomy, breast neoplasms, depression, social support, affective symptoms

## Abstract

Background

Depression, anxiety, or both are common in women with early-stage breast cancer (BC). A relationship is known between low perceived social support (PSS) and depression. We aimed to investigate the relationships between alexithymia, PSS, and Beck Depression Inventory (BDI) score in patients diagnosed with early-stage BC.

Materials and methods

A demographic and medical information form, BDI, the 20-item Toronto Alexithymia Scale (TAS-20), and the Multidimensional Scale of PSS (MSPSS) were given to 200 early-stage BC patients to respond. Two subgroups were created as group A (BDI score < 17) and group B (BDI score ≥ 17) and compared in terms of sociodemographic characteristics, TAS-20, and MSPSS scores.

Results

Twenty-six (18.1%), 48 (33.3%), and 26 (18.1%) patients were with high BDI scores, in low PSS status, and alexithymic, respectively. The median ages of the participants in group A and group B were 53.4 (interquartile range (IQR): 46-60.7) and 46 (IQR: 41.5-59) years, respectively (p = 0.083). The rates of single participants (26.9% versus 11%, p = 0.055), alexithymic participants (42.3% versus 12.7%, p = 0.001), low PSS levels (57.7% versus 28%, p = 0.018), psychiatric treatment history (46.2% versus 22%, p = 0.025), and patients with low income (57.7% versus 22.9%, p = 0.001) were higher in group B than in group A. In the multivariate regression model that contains the parameters mentioned above, psychiatric treatment history (odds ratio (OR): 2.758, 95% confidence interval (CI): 1.034-7.356, p = 0.043), low-income status (OR: 3.503, 95% CI: 1.336-9.182, p = 0.011), and alexithymia (OR: 3.482, 95% CI: 1.229-9.867, p = 0.019) were independent predictive factors for a high BDI.

Conclusion

Alexithymia and low PSS are significantly common in patients with prominent depressive symptoms in early-stage BC patients. Alexithymia may be associated with depression and may also have a role in depression pathogenesis in early-stage BC patients. New studies are needed to investigate whether there is a causal relationship between alexithymia and depression.

## Introduction

Breast cancer (BC) is the most commonly diagnosed cancer and the second most common cause of cancer-related death in women [1]. Of patients diagnosed with breast cancer, 94% are at the non-metastatic stage at diagnosis [2]. The primary treatment of non-metastatic disease is surgical resection with or without adjuvant or neoadjuvant treatment modalities such as chemotherapy, hormonotherapy, and radiotherapy [3]. Approximately 50% of women with early-stage BC had depression, anxiety, or both within the first year after diagnosis, which decreased in the following years [4]. In a study including patients with early-stage BC, depression scores were higher in patients who underwent mastectomy than in those who underwent breast-conserving surgery [5]. Numerous studies have shown that the frequency of depression in BC patients on adjuvant chemotherapy (ACTx) is higher than in those not receiving chemotherapy [6,7].

The concept of alexithymia means “lack of words for emotions”; emotional functioning is relatively narrowed, and the poverty of fantasy life and the inability to find the appropriate words to describe one’s feelings are at the forefront [8,9]. The inability to identify or be aware of emotions can sometimes be confused with an unwillingness to verbalize emotions, even when well aware. Alexithymia can often accompany many psychiatric disorders, such as substance abuse, eating abnormalities, somatoform disorder, depression, and anxiety disorders [9,10]. Higher alexithymia scores have been observed in cancer patients diagnosed within the last three months than in patients with a long history of cancer or healthy individuals [11]. Numerous studies have shown an association between low perceived social support (PSS) and alexithymia in healthy participants [12,13]. Also, high emotional and social support was associated with less depression and distress in early-stage BC patients [14,15]. Boinon et al. showed a relationship between adverse emotional outcomes with negative social support and emotional sharing in alexithymic cases in BC patients after surgery [16].

In our study, we aimed to investigate the relationships between alexithymia, PSS, and Beck Depression Inventory (BDI) score in patients diagnosed with early-stage BC who had mastectomy within the last year and were receiving ACTx we considered to be at high risk for depression [4-7].

## Materials and methods

Study design

This study was a cross-sectional observational descriptive study. It was conducted in the chemotherapy unit of the medical oncology department of a tertiary referral center. The study data was collected between December 2019 and March 2020, after the local ethics committee approval (document number: 2019-12 / 474, date: December 4, 2019).

Study population

Female patients diagnosed with stage I-III BC were included in the study. Being within the first year of diagnosis, treated with radical mastectomy, and on chemotherapy were considered negative risk factors for depression [4-7]. Patients within the first year of diagnosis who underwent a radical mastectomy and were receiving ACTx were included in the study. All patients were literate, were over 18 years of age, had a Karnofsky performance score of at least 80, and had no physical or mental disability. Patients with severe and uncontrolled comorbidities (i.e., heart failure, chronic obstructive pulmonary disease, neurological disease, liver failure, or kidney failure) were excluded. Similarly, patients diagnosed with psychotic disorders were excluded from the study. Thus, a homogeneous group that we expect to have a high risk of depression associated with BC or cancer treatments was obtained. A demographic and medical information form, BDI, the 20-item Toronto Alexithymia Scale (TAS-20), and the Multidimensional Scale of Perceived Social Support (MSPSS) were given to patients to respond. A total of 200 patients were asked to complete the study scales given to them in printed form.

Instruments

Demographic and Medical Information Form

A constructed demographic information form included questions about age, marital status (single or married), place of residence, comorbidities (i.e., diseases such as hypertension, diabetes mellitus, osteoarthritis, and migraine), educational time (total years spent for education at school), income level (what patients report as low, medium, or high, in their own opinion), employment status, and history of psychiatric treatment (i.e., current or past treatments for depression and anxiety).

Beck Depression Inventory

The Beck Depression Inventory consists of 21 questions, each scoring between 0 and 3 [17]. High scores on this scale indicate an increase in the severity of depressive complaints. In the study of Hisli evaluating the validity and reliability of the Turkish version of the BDI, it was observed that a scale score of 17 and above was a level that determined depression above normal, which can reflect clinical depression [18].

Toronto Alexithymia Scale

The Toronto Alexithymia Scale is a self-reporting scale comprising 20 items rated on a 5-point Likert scale ranging from 1 (strongly disagree) to 5 (strongly agree) [19,20]. The TAS-20 consists of three subscales: Difficulty Identifying Feelings (TAS-DIF), Difficulty Describing Feelings (TAS-DDF), and Externally Oriented Thinking (TAS-EOT). TAS-DIF, TAS-DDF, and TAS-EOT subscales consist of seven (score range: 7-35), five (score range: 5-25), and eight (score range: 8-40) items, respectively. A score of 61 or above on the TAS-20 indicates the presence of alexithymia. Güleç et al. demonstrated that the Turkish version of the TAS-20 was valid and reliable for the Turkish sample [21].

Multidimensional Scale of Perceived Social Support

The 12-item Multidimensional Scale of Perceived Social Support (MSPSS) evaluates perceived social support from three sources: family, friends, and significant others. Each item is scored from 1 point (very strongly disagree) to 7 points (very strongly agree). A higher total score indicates higher perceived social support [22]. Zimet et al. divided PSS into three equal groups based on participants’ scores and defined the lowest group as “low perceived support,” the middle group as “moderate support,” and the highest group as “high support” (https://gzimet.wixsite.com/mspss). Eker et al. confirmed the validity and generalizability of the factor structure of the Turkish version of the MSPSS scale [23].

Ethical considerations

This study involved human participants and was approved by the Ethics Committee of the University of Health Sciences (UHS), Dr. Abdurrahman Yurtaslan Ankara Oncology Training and Research Hospital (document number: 2019-12 / 474, date: December 4, 2019). Patients gave informed consent to participate in the study before enrolment. The study has been performed under the ethical standards of the Declaration of Helsinki.

Statistical analysis

Statistical analysis was performed using the Statistical Package for the Social Sciences (SPSS) software (SPSS for Windows, version 24.0., IBM Corp., Armonk, NY, USA). The normality of the distribution of numerical data was evaluated using the Kolmogorov-Smirnov test. Numerical data were presented as median (interquartile range (IQR)), and categorical data were presented as frequency (percentage). The patients were divided into two subgroups: group A (BDI score < 17) and group B (BDI score ≥ 17). Groups A and B were compared using Pearson’s chi-square and Mann-Whitney U tests for categorical and numerical data. Multivariate logistic regression analysis was performed using variables with a p-value below 0.05 due to univariate analysis to determine independent factors predicting high BDI scores. All statistical tests were two-tailed, and p-values < 0.05 were considered statistically significant.

## Results

Two hundred patients who met the inclusion criteria were given an informed consent form to participate in the study. Twenty-four patients answered the questionnaires incompletely, 16 did not want to participate in the study, 13 gave unreliable answers (same answers for all of the questions), and three denied consent. Thus, 56 patients were excluded from the study. A total of 144 female patients with early-stage BC who were surgically treated with total mastectomy and receiving ACTx were included in the statistical analysis. The median age of the patients was 51.3 (IQR: 44.5-60.6) years. Of the patients, 124 (86.1%) were married, and 20 (13.9%) were single. The median educational time (total time spent in school) was five (IQR: 5-11) years. One hundred nineteen (82.6%) patients were unemployed, and 25 (17.4%) were employed. Forty-two (29.2%) patients had low income, and 102 (70.8%) had medium/high income. Fifty-nine (41%) and 85 (59%) patients lived in the country and urban areas, respectively. One hundred sixteen (80.6%) patients had comorbidities, and 38 (26.4%) had a history of psychiatric treatment. The median BDI, TAS-20, and MSPSS scores were 8 (IQR: 5-13.75), 50 (IQR: 43.25-57), and 68 (IQR: 50-79.75), respectively. There were 26 (18.1%) patients with high BDI scores (BDI score ≥ 17). Twenty-six (18.1%) patients were alexithymic. Forty-eight (33.3%) patients were in low PSS status. The BDI, TAS-20, and MSPSS scores and BDI score status, the ratio of alexithymic individuals, and the PSS status of all patients are shown in Table 1.

**Table 1:** Study scale scores, Beck Depression Inventory score status, the ratio of alexithymic individuals, and perceived social support status of all study participants IQR: interquartile range; BDI: Beck Depression Inventory; TAS-20: Toronto Alexithymia Scale; TAS-DIF: Toronto Alexithymia Scale-Difficulty Identifying Feelings; TAS-DDF: Toronto Alexithymia Scale-Difficulty Describing Feelings; TAS-EOT: Toronto Alexithymia Scale-Externally Oriented Thinking; ε: TAS-20 score = or > 61; MSPSS: Multidimensional Scale of Perceived Social Support

Parameter	Median (IQR)
BDI score	8 (5-13.75)
BDI score status	
Low BDI, number (%)	118 (81.9)
High BDI, number (%)	26 (18.1)
TAS-20, total score	50 (43.25-57)
TAS-DIF	16 (12-19)
TAS-DDF	12 (10-15)
TAS-EOT	22 (20-24.75)
Alexithymia^ε^	
No, number (%)	118 (81.9)
Yes, number (%)	26 (18.1)
MSPSS, total score	68 (50-79.75)
MSPSS family subscale	27 (22-28)
MSPSS friends subscale	22.5 (16-28)
MSPSS significant other subscale	20 (12-26.75)
Perceived social support	
High and moderate, number (%)	96 (66.7)
Low, number (%)	48 (33.3)

There were 118 (81.9%) patients in group A with a low BDI score (<17) and 26 (18.1%) patients in group B with a high BDI score (≥17). Patients in both groups were similar in terms of many sociodemographic parameters (Table 2). The ratio of psychiatric treatment history was higher in group B than in group A (46.2% versus 22%, p = 0.025), and group B had more low-income patients than in group A (57.7% versus 22.9%, p = 0.001) (Table 2). The median total score of the TAS-20 scale and the median scores of TAS-DIF, TAS-DDF, and TAS-EOT subscales were higher in group B than in group A, with a statistical significance except for TAS-EOT (Table 3). The median total MSPSS scale score and the median MSPSS subscale scores were lower in group B than in group A, with a statistical significance (Table 3). The ratio of alexithymic participants in group B (42.3% versus 12.7%, p = 0.001) and the ratio of participants with low PSS levels (57.7% versus 28%, p = 0.006) was higher than in group A (Table 3).

**Table 2:** Comparison between group A (BDI score < 17) and group B (BDI score ≥ 17) for sociodemographic characteristics BDI: Beck Depression Inventory; IQR: interquartile range; §: according to the patients’ statement

Parameter	Group A (n = 118)	Group B (n = 26)	p-value
Age, median (IQR)	53.4 (46-60.7)	46 (41.5-59)	0.083
Marital status, number (%)			0.055
Single	13 (11)	7 (26.9)	
Married	105 (89)	19 (73.1)	
Place of residence, number (%)			0.829
Country	49 (41.5)	10 (38.5)	
Urban	69 (58.5)	16 (61.5)	
Comorbidity, number (%)			1.0
No	23 (19.5)	5 (19.2)	
Yes	95 (80.5)	21 (80.8)	
Psychiatric treatment history, number (%)			0.025
No	92 (78)	14 (53.8)	
Yes	26 (22)	12 (46.2)	
Educational time, median (IQR)	5 (5-11)	5 (5-11)	0.639
Income status^§^, number (%)			0.001
Low	27 (22.9)	15 (57.7)	
Moderate and high	91 (77.1)	11 (42.3)	
Employment status, number (%)			0.250
Unemployed	95 (80.5)	24 (92.3)	
Employed	23 (19.5)	2 (7.7)	

**Table 3:** Comparison between group A (BDI score < 17) and group B (BDI score ≥ 17) for study scale scores, the ratio of alexithymic individuals, and perceived social support status All scale scores were given as median (interquartile range). BDI: Beck Depression Inventory; TAS-20: Toronto Alexithymia Scale; TAS-DIF: Toronto Alexithymia Scale-Difficulty Identifying Feelings; TAS-DDF: Toronto Alexithymia Scale-Difficulty Describing Feelings; TAS-EOT: Toronto Alexithymia Scale-Externally Oriented Thinking; ε: TAS-20 score = or > 61; MSPSS: Multidimensional Scale of Perceived Social Support

Parameter	Group A (n = 118)	Group B (n = 26)	p-value
TAS-DIF	15 (11-18)	17.5 (14.75-22)	0.006
TAS-DDF	12 (10-14.25)	14.5 (11.75-18.25)	0.002
TAS-EOT	22 (19-24)	23 (20-27.25)	0.237
TAS-20, total score	49 (42-56)	56.5 (48.5-62.5)	0.002
Alexithymia^ε^			0.001
No, number (%)	103 (87.3)	15 (57.7)	
Yes, number (%)	15 (12.7)	11 (42.3)	
MSPSS family subscale	28 (23.75-28)	22 (17.75-28)	0.003
MSPSS friends subscale	25 (17-28)	16 (5.75-22.25)	<0.001
MSPSS significant other subscale	21 (14-27)	16.5 (8.5-23.25)	0.043
MSPSS, total score	70.5 (54-80.25)	50.5 (35.75-72)	0.001
Perceived social support			0.006
Low, number (%)	33 (28)	15 (57.7)	
High and moderate, number (%)	85 (72)	11 (42.3)	

The binary logistic regression analysis results, which include the factors that can predict a high BDI score, are shown in Table 4. Psychiatric treatment history (odds ratio (OR): 2.758, 95% confidence interval (CI): 1.034-7.356, p = 0.043), low-income status (OR: 3.503, 95% CI: 1.336-9.182, p = 0.011), and alexithymia (OR: 3.482, 95% CI: 1.229-9.867, p = 0.019) were independent predictive factors for a high BDI.

**Table 4:** Logistic regression analysis results including factors that can predict a high Beck Depression Inventory score OR: odds ratio; SE: standard error; CI: confidence interval; PSS: perceived social support

Independent variables	OR	SE	95% CI		p-value
			Lower	Upper	
Psychiatric treatment history (yes versus no)	2.758	0.501	1.034	7.356	0.043
Income status (low versus moderate and high)	3.503	0.492	1.336	9.182	0.011
Alexithymia (yes versus no)	3.482	0.531	1.229	9.867	0.019
PSS (low versus high)	2.218	0.495	0.841	5.852	0.107

## Discussion

In our study, we evaluated the factors that may predict high BDI scores in patients with BC who had undergone a radical mastectomy, were diagnosed within the last year, and received ACTx, which we considered high risk for depression. Although the BDI score alone is not sufficient for diagnosing depression, a BDI score of 17 and above have been shown to have clinical significance in depression [18]. The rates of alexithymia and low PSS were higher in patients with high BDI scores than in patients with low BDI scores. In our multivariate regression model, psychiatric treatment history, low-income status, and alexithymia were independent predictive factors for a high BDI score.

Patients diagnosed with cancer present many psychiatric symptoms and diseases, especially depression, depending on cancer and cancer treatment [24,25]. In a recent study, a significant portion of patients with BC was depressed (38.2%) and anxious (32.2%) [26]. Even at an early stage, patients diagnosed with BC are at risk for depression, especially in the first year of diagnosis [4]. In our study, 18.1% of our patients had a high BDI score, and it can be said that these patients have significant symptomatology in terms of depression.

Although there is no clear consensus on whether alexithymia should be considered a trait or a state, Freyberger suggested that secondary alexithymia triggered by a traumatic event such as a life-threatening disease may accompany primary alexithymia [27]. The large-scale study of Gürsoy et al., which was conducted in the COVID-19 pandemic environment, has suggested that individuals with alexithymic features are more sensitive to stress during health-related increased stress [28]. Moreover, the relationship between low PSS and alexithymia [12,13] and high emotional support with low depression and distress [14,15] should also be kept in mind. Therefore, it may be wise to consider alexithymia and PSS together when evaluating depression in cancer patients, especially during the early treatment period.

In the review by De Vries et al., including 16 studies on alexithymia in cancer patients, only four examined the relationship between alexithymia and psychopathology [29]. Three [30-32] of these four studies showed a relationship between alexithymia and psychopathology in cancer patients, but one study [33] failed to show any relationship. In a well-designed study in which 105 patients diagnosed with BC were included, scores on the Quality of Life Scale dimensions indicated more depression (t = 5.58, df = 103, p < 0.001) and poorer well-being (t = 3.38, df = 103, p < 0.01) for patients meeting the Diagnostic Criteria for Psychosomatic Research cluster of alexithymia [30]. The analysis performed with 46 postsurgical ambulatory women with BC and their husbands revealed a correlation between a high degree of alexithymia and a high degree of anxiety in both patients and their husbands [31]. The study by Luminet et al., in which they evaluated the temporal variation of depression and alexithymia in 122 BC patients, revealed that depression was correlated with total alexithymia scores both at baseline (r = 0.32, p < 0.001) and at follow-up (r = 0.62, p < 0.001) [32]. The authors support the view that the relative stability of alexithymia is a stable personality trait rather than a state-dependent phenomenon [32]. In our study, the median TAS-DIF, TAS-DDF, and TAS-20 total scores and the rate of alexithymic individuals were significantly higher in the group with a high BDI score than in the group with a low BDI score. Also, alexithymia was an independent predictive factor for a high BDI score in our multivariate regression model. However, it should be noted that the probability of this prediction may vary in different multivariate regression models. The TAS-20 scale, which we use in assessing alexithymia, actually brings a paradox. The fact that a “word”-based measurement method was used to evaluate the absence of “word” can be considered a limitation. However, it is also useful because it provides objectivity for obtaining numerical values and statistical analysis. The current literature data and our study findings suggest a relationship between alexithymia and depression. Although our study did not investigate causality, our findings suggested that alexithymia may play a role in the etiopathogenesis of depression in patients with early-stage BC.

Two decades ago, Alferi et al. drew attention to an inverse relationship between distress and perceptions of partner support in patients with early-stage BC [14]. Although this study is valuable due to its longitudinal design, only correlation analysis was performed, and possible relationship mediators/effectors were not considered [14]. Similarly, many studies have revealed a relationship between PSS and depression in patients with BC [15,34,35]. The relationship between alexithymia and PSS has been demonstrated in many different populations, such as late adolescents, patients followed up with the diagnosis of fibromyalgia, and physicians working in palliative care [13,36,37]. It may be a reasonable method to consider alexithymia with PSS in evaluating depression. As far as we know, only one study in the literature by Boinon et al., conducted in the French Cancer Center Gustave Roussy Institute, evaluated all of these concepts together [16]. Boinon et al. evaluated patients with early-stage, surgically treated BC (N = 113) in two groups, a low-alexithymia group (n = 55) and a high-alexithymia group (n = 58) [16]. In the high-alexithymia subgroup, higher depressive symptoms were associated with higher self-reported negative support (β = 0.426, p < 0.01), but not in the low-alexithymia group [16]. A significant difference in the role of negative support on depression in the high-alexithymia group between the low-alexithymia group (z = -3.29, p < 0.01) was considered favoring a moderator effect of alexithymia on the negative support-depression link [16]. The most important finding of our study is that alexithymia is an independent predictive factor for a high BDI score. However, although PSS scores were significantly lower in the high BDI group than in the low BDI group, PSS was not an independent predictive factor for a high BDI in multivariate analysis. Our findings agree with those of Boinon et al. The relationship between low PSS and alexithymia is likely due to alexithymia-related deficiencies in social skills [38]. Therefore, it can be said that PSS is low in patients with early-stage BC who develop depressive symptoms, and alexithymia may be the primary mediator of this low level.

Early-stage BC patients occupy a vast place in oncology practice, but our study results may not reflect all cancer patients. Therefore, the relationship between alexithymia, PSS, and depression should also be evaluated in patients with different cancer types or stages. Although income level and psychiatric treatment history were independent predictive factors for a high BDI in our study, our study was not designed to evaluate these factors in the context of the relationship between alexithymia, PSS, and depression. Our study findings may also show time-dependent changes; it may be appropriate to conduct a new study similar to our current study with a longitudinal design. Psychological interventions targeting alexithymic individuals have shown positive contributions to pain control in cancer patients and in improving the well-being of asthma patients [39,40]. To the best of our knowledge, there is no psychological intervention study targeting alexithymic individuals to improve depression or other psychiatric disorders in cancer patients, and such a study is needed.

## Conclusions

Psychiatric disorders, especially depression, are significant problems in cancer patients, even with early-stage BC. There is a relationship between depressive symptoms, low perceived social support, and alexithymia in early-stage BC patients. Also, alexithymia may have a role in depression pathogenesis in early-stage BC patients. New studies are needed to evaluate the relationship between depression, alexithymia, and perceived social support in various cancer patient groups. Also, there is a need for intervention studies on this relationship and studies to evaluate the relationship in terms of causality.
